# Predicting long-term mortality in hospitalized elderly patients using the new ESPEN definition

**DOI:** 10.1038/s41598-017-04483-1

**Published:** 2017-06-22

**Authors:** Jiaojiao Jiang, Xiaoyi Hu, Jing Chen, Haozhong Wang, Lei Zhang, Birong Dong, Ming Yang

**Affiliations:** 1 The Center of Rehabilitation, West China Hospital, Sichuan University, No.37 Guoxue Lane, Chengdu, Sichuan China; 2 The Center of Gerontology and Geriatrics, West China Hospital, Sichuan University, No.37 Guoxue Lane, Chengdu, Sichuan China; 3 The Department of Orthopedic surgery, West China Hospital, Sichuan University, No.37 Guoxue Lane, Chengdu, Sichuan China

## Abstract

The European Society of Clinical Nutrition and Metabolism (ESPEN) recently published new diagnostic criteria for malnutrition. The aim of this study was to evaluate whether malnutrition by the new ESPEN diagnostic criteria can predict long-term mortality in elderly inpatients. We conducted a prospective study in the acute geriatric wards. Malnutrition was defined according to the new ESPEN criteria and the Mini Nutritional Assessment (MNA), respectively. The survival status was determined by telephone interviews at 3-years. A total of 437 elderly adults were included. According to the new ESPEN criteria, 66 participants (15.1%) were malnourished. According to the MNA, 45 participants (10.3%) were identified as malnourished. The 3-year all-cause mortality was 41.7% in participants with malnutrition defined by the ESPEN criteria and 15.3% in participants without malnutrition (p < 0.001). After adjusting for relevant confounders, malnutrition defined by the ESPEN criteria was a significant predictor of 3-year all-cause mortality (hazard ratio [HR] 2.98, 95% confidence interval [CI] 1.87–4.86). However, malnutrition defined by the MNA was not a significant predictor of 3-year all-cause mortality (HR 1.67, 95% CI 0.89–2.31). In conclusion, the new ESPEN diagnostic criteria for malnutrition are reliable in predicting 3-year all-cause mortality among elderly inpatients.

## Introduction

Malnutrition, also known as undernutrition, can be simply defined as “any nutritional imbalance”^1^. In adults, malnutrition generally occurs when nutrient intake is consistently insufficient to meet individual nutrient requirements, and this imbalance between nutrient intake and requirements ultimately results in changes in body weight, body composition, and physical function^1, 2^. In various study populations, malnutrition is known to be independently associated with various morbidities, mortality, functional impairment, poorer quality of life, and increased healthcare costs^1, 3–6^.

The prevalence of malnutrition increases with age, the number of comorbidities, and level of care^4, 7^. It is prevalent in elderly people, especially in hospitalized elderly patients^4, 7^. In previous studies, the prevalence of malnutrition in elderly inpatients ranged from approximately 20 to 60%^3, 4, 8^. Fortunately, early detection and interventions for malnutrition (e.g., oral nutritional supplements or dietary counseling) in elderly inpatients can significantly improve their major clinical outcomes, such as mortality and quality of life^5, 7, 9, 10^. Therefore, nutritional status screening is currently recommended as a routine process across the continuum of care, especially in the acute care setting^8, 10^.

Although malnutrition has been extensively studied for decades, there are no clearly accepted diagnostic criteria for malnutrition. In order to address this issue, the European Society of Clinical Nutrition and Metabolism (ESPEN) recently published a new consensus statement on the diagnosis of malnutrition, which includes a two-step process. First, a validated risk screening tool is recommended to identify individuals “at risk of malnutrition”. Second, in those who are at risk of malnutrition, two alternative ways are offered to diagnose malnutrition: 1) body mass index (BMI) <18.5 kg/m^2^; or 2) unintentional weight loss >10% indefinite of time (or >5% over the last 3 months) combined with either of the following two items: BMI <20 kg/m^2^ if age <70 years or <22 kg/m^2^ if age ≥70 years; or low fat-free mass index (FFMI) <15 and 17 kg/m^2^ in women and men, respectively^11^. This new definition of malnutrition needs to be validated in specific populations. Therefore, we conducted this study 1) to determine the prevalence of malnutrition among hospitalized elderly inpatients according to the new ESPEN diagnostic criteria; and 2) to evaluate whether malnutrition by the new ESPEN diagnostic criteria can predict long-term mortality in these patients.

## Methods

This study is a post-hoc analysis of a prospective study that was conducted in the acute geriatric wards of the West China Hospital of Sichuan University and the Fifth People’s Hospital of Chengdu City. The study protocol was approved by the Research Ethics Committee of Sichuan University. All methods in this study were in accordance with relevant guideline and regulations. A written informed consent was signed by all participants or their legal proxies.

### Participants in baseline investigation

During August to December 2012, consecutively admitted elderly patients (aged 60 years or older) in the acute geriatric wards of the two hospitals were invited to participate in this study. Patients who could not finish the face-to-face interviews and/or anthropometric measurements due to severe health problems were excluded. They generally had at least one of the following conditions: 1) diseases in the terminal stage; 2) severe cognitive impairment; and 3) delirium. In addition, patients with clinically visible edema were also excluded.

### Data collection

The main baseline data were collected by trained interviewers through face-to-face interviews within 48 hours after admission. Additionally, three trained technicians performed the following anthropometric measurements: body weight, height, calf circumference (CC), waist circumference (WC), and mid-arm circumference (MAC). We used a wall-mounted stadiometer and a digital floor scale to measure body height and weight to the nearest 0.1 cm and 0.1 kg, respectively. BMI was calculated as the ratio between weight (kg) and squared height (m) (kg/m^2^). We measured the calf circumference at its widest point using a flexible tape to the nearest 0.1 cm when the participant placed in the supine position, with the left knee raised and the calf placed at a right angle to the thigh. We measured MAC using a flexible tape to the nearest 0.1 cm at the mid-point between the tip of the acromion and the olecranon process of the left upper arm. WC was measured using a flexible tape at the top of the hip bone to the nearest 0.1 cm on the naked skin at the end of light exhalation with the subject standing. A preliminary study was conducted to assess the reliability of the anthropometric measurements using the intraclass correlation coefficient (ICC). The results showed an excellent test-retest reliability of the anthropometric measurements (ICC = 0.82; n = 90).

### Nutritional status assessment

According to the new ESPEN criteria^11^, two steps were used to identify malnutrition (marked as malnutrition _ESPEN_ in this study). First, the nutritional status of each participant was assessed using the Mini Nutritional Assessment Short-Form (MNA-SF)^12^. A score of ≤11 indicated that a participant was “at risk of malnutrition”. Second, in the participants who were at risk of malnutrition by the MNA-SF, the diagnosis of malnutrition was confirmed according to either of the following criteria: 1) BMI <18.5 kg/m^2^; and 2) unintentional weight loss >10% indefinite of time (or >5% over the last 3 months) combined with BMI <20 kg/m^2^ if age <70 years or <22 kg/m^2^ if age ≥70 years.

In addition, the nutritional status of each participant was also assessed using the full version of the Mini Nutritional Assessment (MNA). Participants with scores of less than 17 were classified as malnutrition (marked as malnutrition _MNA_ in this study).

### Covariates

We used the Older Americans Resources and Services (OARS) multidimensional functional assessment questionnaire^13^ to assess the activities of daily living (ADLs) and instrumental activities of daily living (IADLs). The response to each item was “without help” (1 point), “with some help” (2 points) and “completely unable to do” (3 points). The validity and reliability of the two scales have been well established^13–15^. The subjects were considered having an ADL or IADL disability when they reported a need for help from another person in performing at least one ADL item (ADL disability) or one IADL item (IADL disability)^13–15^.

Depression was measured using the Chinese version of the 30-item Geriatric Depression Scale (GDS-30)^16^, and a score of ≥11 suggests depression^16^. Cognitive function was assessed using the previously validated Chinese version of the Mini-Mental Status Examination (MMSE)^17^. The diagnostic cut-off points for cognitive impairment are as follows: a score of ≤17 for less than primary school, ≤20 for primary school graduates, and ≤24 for high school graduates or those with higher education^18^.

The following covariates were also collected from the face-to-face interviews: age, sex, education level, physical activity, alcohol drinking status, and smoking status. The following comorbidities were identified according to hospital information systems: hypertension, diabetes, obstructive pulmonary disease (COPD), ischemic heart disease, stroke, chronic kidney disease, acute infection, cancer of any type, osteoarthritis, liver disease, gastrointestinal disease, urinary incontinence, and chronic pain. The information about nutritional supplements during hospitalization was also collected. Handgrip strength was also measured by trained technicians using a handheld dynamometer based on strain gauge sensors (EH101, Xiangshan Inc., Guangdong, China) to the nearest 0.1 kg. In addition, serum prealbumin and hemoglobin were measured.

### Follow-up

The survival data of the participants were obtained by telephone interviews at 12, 24, and 36 months during the 3-year follow-up period. These data were also confirmed using the Local Death Registry Database. Time to death was calculated as the time between the first interview and the date of death to the nearest one month.

### Statistical analysis

All statistical analyses were performed using SPSS 20.0 (IBM SPSS Statistics, Armonk, NY, USA). The categorical data and continuous data were presented as absolute numbers and percentages (%), and median and interquartile range (IQR), respectively. To compare the differences between groups, we used the Pearson chi-squared test for categorical data and the Mann-Whitney U test for continuous data with abnormal distribution, respectively. A p value of <0.05 was considered statistically significant. Univariate Cox proportional hazard analysis was used to investigate the possible predictors of 3-year all-cause mortality. The results were presented as hazard ratio (HR) and 95% confidence interval (CI). In order to explore the independent risk factors of mortality, we also performed multivariable Cox regression models. Variables that exhibited a significant association in univariate analysis were considered to enter in the first step of multivariable Cox regression models. However, in order to minimize the effect of collinearity, CC and MAC were excluded from the multivariable Cox regression models. In addition, survival curves were estimated using the Kaplan–Meier method and compared using log-rank tests.

## Results

### Characteristics of the study population

**Figure 1:**
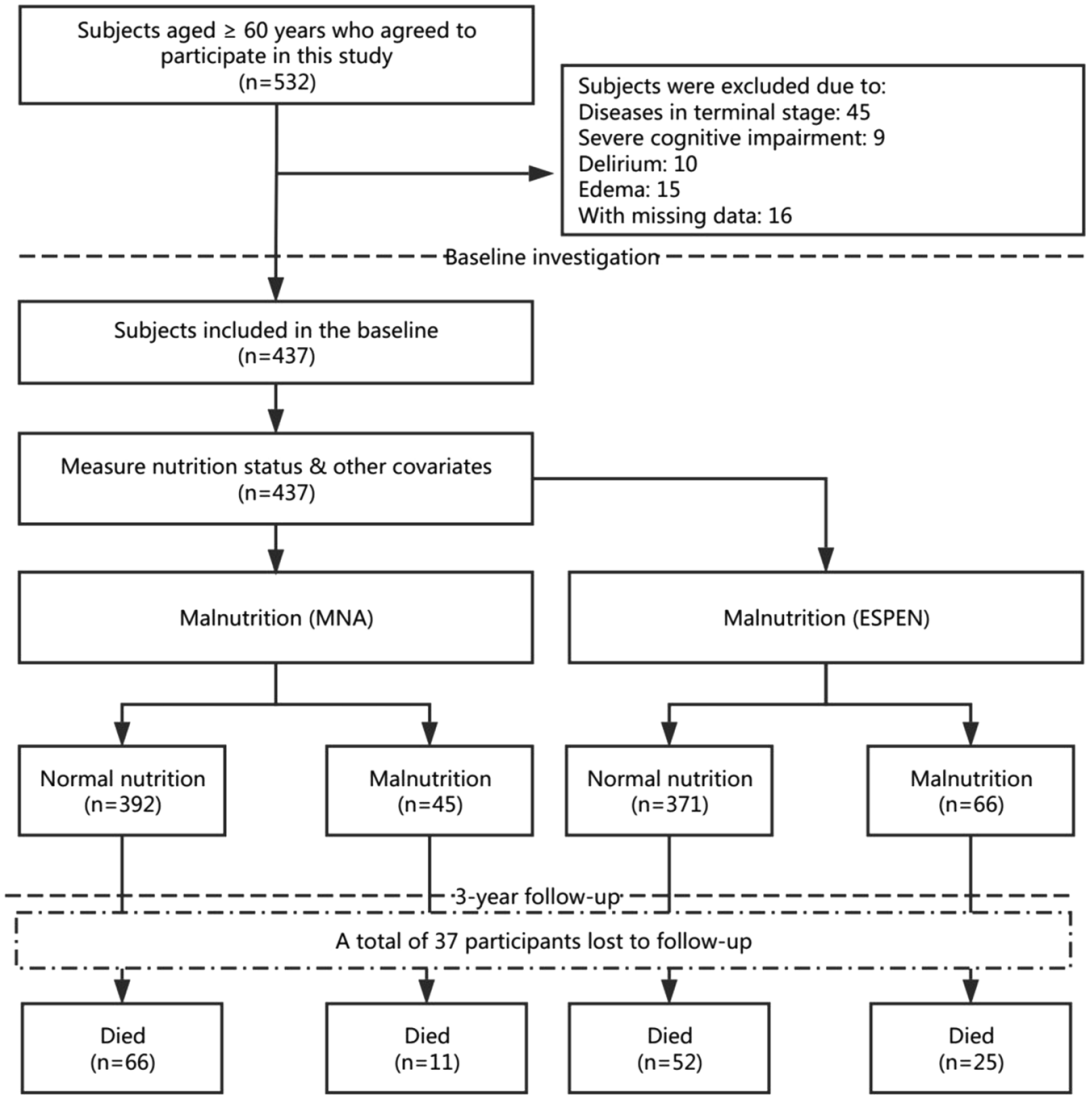
The flowchart of the study design.

Among the 532 individuals who agreed to join this study, 79 individuals were excluded (Fig. 1). In addition, data needed to assess malnutrition were missing in 16 participants (3%). As a result, 437 participants were included in the baseline analyses. There was no significant difference between the included subjects and the excluded subjects with respects to age (median age: 81.0 vs. 80.5 years, p = 0.867) and gender (women: 29.5% vs. 31.6%, p = 0.691). During the 3-year follow-up, 37 participants (8.5%) were lost to follow-up, which led to a final population of 400 participants, of which, 77 participants (19.3%) died.

Compared with the survivors, the deceased participants were significantly older (median age: 82.0 years versus 81.0 years, p = 0.001), and more prone to suffer from tumor (18.2% versus 9.6%, p = 0.032), ADL disability (51.9% versus 31.6%, p < 0.001), IADL disability (74.0% versus 48.6%, p < 0.001), depression (42.5% versus 13.0%, p < 0.001), cognitive impairment (37.7% versus 19.2%, p < 0.001), and lower hemoglobin (median: 101.0 g/L versus 124.0 g/L, p = 0.006) (Table 1).

**Table 1:** Baseline characteristics of the whole study population and stratified by survivors and deceased at the end of a 3-year follow-up^a^.

Characteristic	Total of baseline(n = 437)	Survivors (n = 323)	Deceased (n = 77)	p-value
Age (years)*	81.0 (74.5–84.0)	81.0 (74.0–83.0)	82.0 (79.0–87.0)	0.001
Women (%)	129 (29.5)	99 (30.7)	15 (19.5)	0.051
Current smokers (%)	53 (12.1)	40 (12.4)	8 (10.4)	0.193
Current alcohol drinkers (%)	52 (11.9)	41 (12.7)	6 (7.8)	0.230
Physical activity ≥ 30 min/d (%)	245 (56.1)	185 (57.3)	50 (51.9)	0.397
Comorbidities (%)				
Hypertension	241 (55.1)	186 (57.6)	38 (49.4)	0.191
Ischemic heart disease	125 (28.6)	92 (28.5)	25 (32.5)	0.490
COPD	137 (31.4)	98 (30.3)	27 (35.1)	0.422
Diabetes	114 (26.1)	83 (25.7)	23 (29.9)	0.456
Stroke	28 (6.4)	19 (5.9)	54 (5.2)	0.816
CKD	56 (12.8)	39 (12.1)	14 (18.2)	0.155
Acute infection	123 (28.1)	87 (26.9)	25 (32.5)	0.331
Osteoarthritis	111 (25.4)	84 (26.0)	20 (26.0)	0.995
Tumor of any type	47 (10.8)	31 (9.6)	14 (18.2)	0.032
GI disease	84 (19.2)	65 (20.1)	12 (15.6)	0.364
Liver disease	34 (7.8)	24 (7.4)	6 (6.5)	0.776
Urinary incontinence	47 (10.8)	30 (9.3)	11 (14.3)	0.194
Chronic pain	114 (26.1)	86 (26.6)	22 (28.6)	0.730
MNA-SF scores *	11.0 (9.0–13.0)	12.0 (9.5–13.0)	10.0 (8.0–12.0)	<0.001
MNA scores *	24.0 (18.5–26.0)	25.0 (19.0–26.0)	22.0 (16.5–24.0)	<0.001
Malnutrition _MNA_ (%)	45 (10.3)	30 (9.3)	12 (15.6)	0.105
Malnutrition _ESPEN_ (%)	66 (15.1)	35 (10.8)	25 (32.5)	<0.001
Nutritional supplements (%)	48 (11.0)	38 (11.9)	6 (7.8)	0.302
BMI (cm) *	22.4 (19.8–24.7)	22.7 (20.2–25.1)	19.8 (18.4–23.2)	<0.001
CC (cm) *	33.0 (30.0–35.0)	33.0 (30.0–35.7)	32.0 (28.8–35.0)	0.119
WC (cm) *	87.0 (81.0–96.1)	88.0 (82.0–96.0)	86.0 (81.0–97.0)	0.305
MAC (cm) *	26.0 (24.0–29.2)	26.0 (24.0–29.0)	26.0 (23.0–29.0)	0.249
Handgrip strength (kg) *	20.2 (14.4–26.2)	20.6 (14.6–26.7)	20.0 (14.5–24.6)	0.154
ADL scores *	7.0 (7.0–8.0)	7.0 (7.0–8.0)	8.0 (7.0–12.5)	<0.001
ADL disability (%)	285 (65.2)	102 (31.6)	40 (51.9)	<0.001
Without ADL disability (%)	152 (34.8)	221 (68.4)	37(48.1)	
IADL scores *	8.0 (7.0–13.0)	7.0 (7.0–13.0)	11.0 (7.0–16.0)	<0.001
IADL disability (%)	207 (47.4)	157 (48.6)	57 (74.0)	<0.001
Without IADL disability (%)	230 (52.6)	166 (51.4)	20 (26.0)	
GDS-30 scores *	7.0 (3.0–10.0)	6.0 (3.0–10.0)	9.0 (7.0–12.5)	<0.001
Depression (%)	66 (15.1)	42 (13.0)	23 (42.5)	<0.001
Without depression (%)	371 (84.8)	281 (87.0)	54 (57.5)	
MMSE scores *	26.0 (22.0–28.0)	26.0 (23.0–29.0)	24.0 (19.0–27.0)	0.001
Cognitive impairment (%)	92 (20.8)	62 (19.2)	29 (37.7)	<0.001
Without cognitive impairment (%)	345 (78.9)	261 (80.8)	48 (62.3)	
Prealbumin (mg/L) *	202.0 (151.5–242.0)	197.5 (153.5–237.8)	207.0 (134.3–235.3)	0.670
Hemoglobin (g/L) *	124.0 (111.0–135.6)	125.0 (113.0–135.0)	117.0 (101.0–136.0)	0.006

*Data are presented as median (IQR). ^a^Thirty-seven participants lost follow-up during the 3-year period.

The chi-square test was performed for categorical data and the Mann–Whitney’s U-test for continuous data with abnormal distribution. P < 0.05 was considered statistically significant.

ADL: activities of daily living; BMI: body mass index; CC: calf circumference; CKD: chronic kidney disease; COPD: chronic obstructive pulmonary disease; GDS-30: 30-item Geriatric Depression Scale; GI: gastrointestinal; IADL: instrumental activities of daily living; IQR: interquartile range; MAC: mid-arm circumference; MMSE: Mini-Mental Status Examination; MNA-SF: Mini Nutritional Assessment Short-Form; WC: waist circumference.

### Prevalence of malnutrition using different criteria

**Figure 2:**
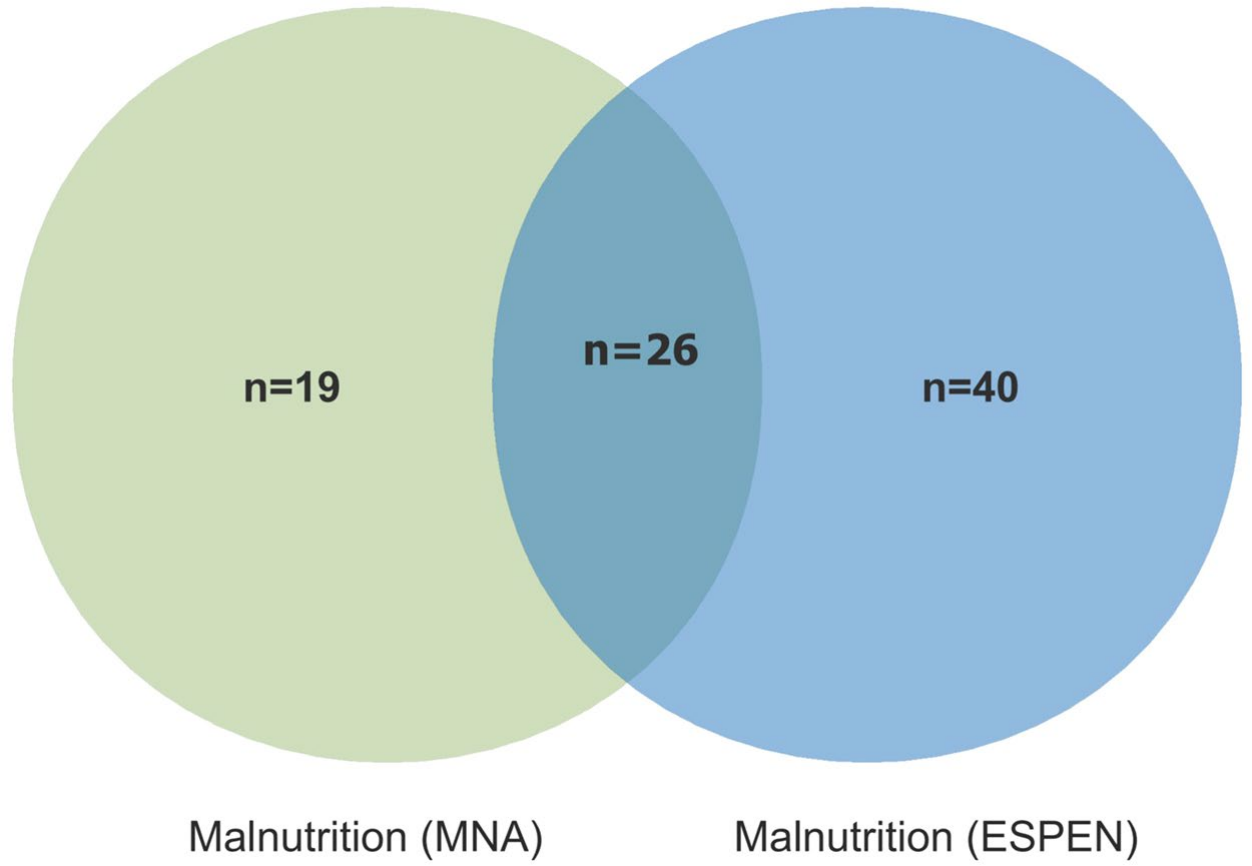
Venn diagram illustrating the overlap between malnutrition _MNA_ and malnutrition _ESPEN_.

According to the MNA, 45 participants (10.3%) in the baseline investigation were identified as malnutrition _MNA_. According to the new ESPEN definition of malnutrition, the prevalence of malnutrition _ESPEN_ was 15.1% (66/437) in the baseline investigation. The overlap between malnutrition _MNA_ and malnutrition _ESPEN_ is illustrated in Fig. 2.

In addition, the prevalence of malnutrition _MNA_ was similar between the final study group and the group lost to follow-up (10.5% vs. 8.1%, p = 0.647). Similarly, no significant difference was identified between these two groups with respect to the prevalence of malnutrition _ESPEN_ (15.0% vs. 16.2%, p = 0.843).

Compared with the survivors, the decreased participants were more prone to have malnutrition _MNA_, but the difference was not statistically significant (15.6% versus 9.3%, p = 0.105). However, the prevalence of malnutrition _ESPEN_ was significantly higher in the decreased group than in the survival group (32.5% versus 10.8%, p < 0.001) (Table 1).

### Association between malnutrition _MNA_ and mortality

**Figure 3:**
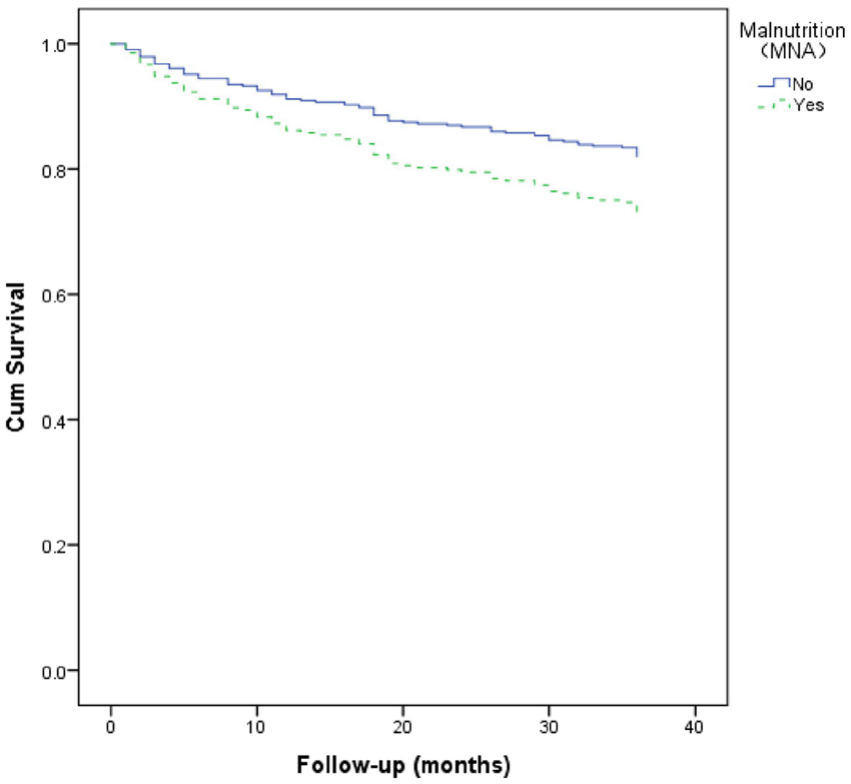
Survival curves of the study population according to malnutrition _MNA_ at baseline. Survival curves did not significantly differ in the log-rank test (p = 0.094).

The 3-year all-cause mortality was 26.2% in participants with malnutrition _MNA_ and 18.4% in participants without malnutrition _MNA_ (p = 0.228). Table 2 shows the predictors of 3-year all-cause mortality according to Cox proportional hazard regression models. Malnutrition _MNA_ was not a significant predictor of 3-year all-cause mortality (HR 1.67, 95% CI 0.89–2.31). However, age (HR 1.07, 95% CI 1.02–1.12), tumor of any type (HR 1.63, 95% CI 1.13–3.06), and cognitive impairment (HR 2.00, 95% CI 1.16–3.45) were significant predictors of 3-year all-cause mortality. In addition, the survival curves of the participants categorized by malnutrition _MNA_ are presented in Fig. 3. These survival curves were not significantly different by the log-rank test (p = 0.094).

**Table 2:** Predictors of 3-year all-cause mortality according to Cox proportional hazard regression models.

Variables	Univariate model^a^			Multivariate model^b^			Multivariate model^c^		
	HR	95% CI	P	HR	95% CI	P	HR	95% CI	P
Malnutrition _MNA_	1.61	0.92–2.83	0.098	1.67	0.89–2.31	0.073	−	−	−
Malnutrition _ESPEN_	3.18	1.98–5.12	<0.001	—	—	—	2.98	1.87–4.86	<0.001
ADL disability	2.19	1.40–3.43	0.001	1.06	0.56–1.98	0.851	1.23	0.73–2.10	0.432
IADL disability	2.76	1.66–4.59	<0.001	1.54	0.72–3.31	0.266	1.76	0.83–3.72	0.141
Age (years)	1.05	1.03–1.21	<0.001	1.07	1.02–1.12	0.007	1.04	1.01–1.08	0.024
Tumor of any type	2.02	1.13–3.61	0.017	1.63	1.13–3.06	0.028	2.06	1.19–3.55	0.010
Depression	2.34	1.38–3.95	0.002	1.36	0.77–2.40	0.283	1.50	0.79–2.84	0.213
Cognitive impairment	2.17	1.30–3.63	0.003	2.00	1.16–3.45	0.013	1.83	1.23–3.14	0.007

Abbreviations: CI: confidence interval; HR: hazard ratio.

In Cox regression (univariate and multivariate), normal nutritional status, independence in ADL and IADL, lower age, and not having tumor of any type, depression, and cognitive impairment were used as reference categories.

^a^Only significant variables are presented except for malnutrition. ^b^Using malnutrition _MNA_ as a variable. ^c^Using malnutrition _ESPEN_ as a variable.

### Association between malnutrition _ESPEN_ and mortality

**Figure 4:**
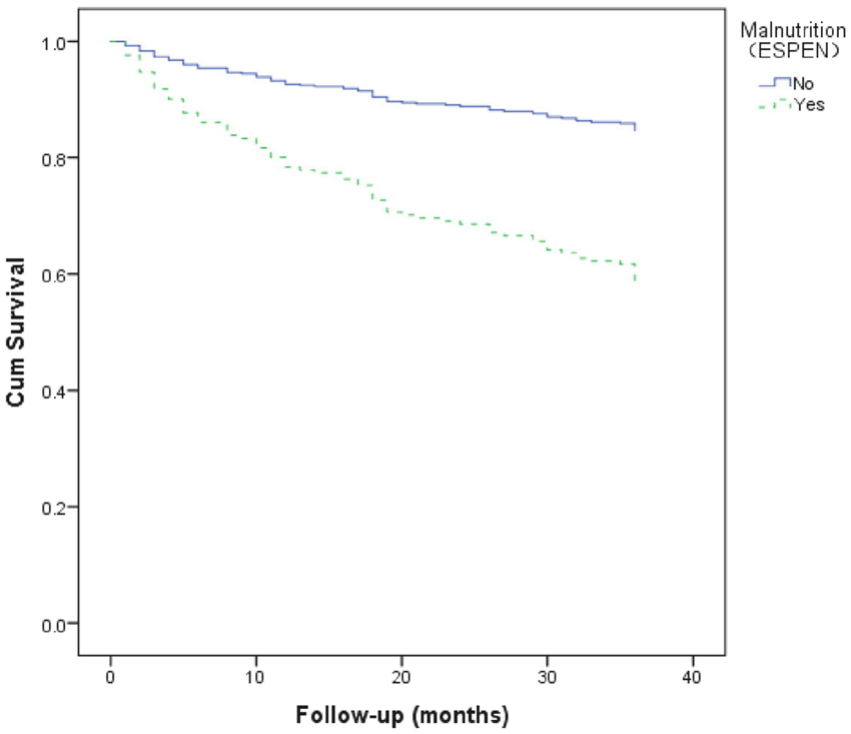
Survival curves of the study population according to malnutrition _ESPEN_ at baseline. Survival curves significantly differed in the log-rank test (p < 0.001).

The 3-year all-cause mortality was 41.7% in participants with malnutrition _ESPEN_ and 15.3% in participants without malnutrition _ESPEN_ (p < 0.001). According to the Cox proportional hazard model, malnutrition _ESPEN_ was a significant predictor of 3-year all-cause mortality (HR 2.98, 95% CI 1.87–4.86). Age (HR 1.04, 95% CI 1.01–1.08), tumor of any type (HR 2.06, 95% CI 1.19–3.55), and cognitive impairment (HR 1.83, 95% CI 1.83, 95% CI 1.23–3.14) were also significant predictors of 3-year all-cause mortality (Table 2). The survival curves of the participants categorized by malnutrition _ESPEN_ are presented in Fig. 4. These survival curves were significantly different by log-rank test (p < 0.001).

## Discussion

The aim of the ESPEN statement of malnutrition is to “provide a general diagnosis that is relevant for all subjects in all clinical settings”^11^. In developed countries, malnutrition occurs primarily in elderly adults^10^. Therefore, it is of great importance to validate the new ESPEN diagnostic criteria of malnutrition in this age group. To the best of our knowledge, this study is the first to validate the new ESPEN diagnostic criteria of malnutrition for the prediction of long-term mortality in elderly inpatients. This study demonstrated that the prevalence of malnutrition was 15.1% and 10.3% based on the new ESPEN diagnostic criteria and the MNA scale, respectively. In our study population, malnutrition defined by the new ESPEN statement is an independent predictor of 3-year all-cause mortality; whereas malnutrition according to the MNA is not.

Because the new ESPEN statement of malnutrition was only recently released, there have been only two published validation studies regarding it^19, 20^. One study investigated the prevalence of malnutrition based on the new ESPEN criteria (with the short nutritional assessment questionnaire as initial screening) in four diverse populations^19^. The prevalence of malnutrition was 14% in acutely ill middle-aged patients, 6% in geriatric outpatients, 0.5% in healthy old individuals, and 0% in healthy young individuals. Another study reported that according to the new ESPEN criteria (with the MNA-SF as initial screening), the prevalence of malnutrition was 6.73% in a population of geriatric inpatients with diabetes^20^. The corresponding prevalence in our study was 15.1%. Because of the significant heterogeneity of study populations, it was hard to make a direct comparison across these studies.

The association between malnutrition and mortality in various populations has been widely studied in previous studies. However, only one study demonstrated the association between the new ESPEN definition of malnutrition and mortality. In their study, Sanz-París *et al*. found that malnutrition defined by the new ESPEN criteria increased 2.7 times the odds of death in the hospital among a population of elderly inpatients with diabetes^20^. Our study adds to this evidence that malnutrition based on the new ESPEN criteria also increases the risk of long-term mortality in elderly inpatients with acute diseases. Further prospective studies are warranted to confirm this relationship in different study populations.

Our study demonstrated that malnutrition assessed by the MNA was not significantly associated with 3-year all-cause mortality. According to a recent systematic review, among the 20 included studies, three studies reported that malnutrition measured by the MNA was not associated with mortality, but there was a significant association in the other 17 studies^21^. The significant heterogeneity between studies made it impossible to combine these results using meta-analyses.

The ESPEN statement of malnutrition recommended that “any validated risk screening tool” could be used for screening malnutrition risk, and this is the first and mandatory step in the diagnosis of malnutrition^11^. In this study, we applied the MNA-SF as the screening tool. In fact, Nutritional Risk Screening 2002 (NRS-2002) and Malnutrition Universal Screening Tool (MUST) were also recommended by the ESPEN statement. The choice of malnutrition risk screening tools appears to affect the results of malnutrition diagnosis. In the future, it will be important to assess the validation of different risk screening tools, and their influence on the diagnosis of malnutrition and healthcare outcomes.

This study has some limitations. First, the second ESPEN diagnostic criterion of malnutrition is based on the combination of unintentional weight loss, and low BMI or low FFMI^11^. However, we did not use the FFMI in this study, because this study is a post-hoc analysis of a prospective study, and no data relevant to FFMI in the study population were available. This might induce bias on the identification of malnutrition. However, it is notable that devices for FFMI measurements, such as bioelectrical impedance analyzers (BIA), are not readily available for clinical practice. Therefore, the ESPEN consensus statement also argued that “it was crucial not to mandate FFMI for the diagnosis of malnutrition”^11^. Second, although we found that nutritional supplements were equivalent between the survivor group and the decreased group, we did not collect the information about nutritional supplements after discharge. This might induce a bias to the results, as there is a growing evidence that nutritional interventions (e.g., individualized nutritional care by dietitians, nutritional supplements, post-discharge home visits, and/or telephone follow-ups) in elderly patients could improve their nutritional and functional status, and mortality rates^22, 23^. Third, we excluded 95 patients in the final analyses, this might induce a bias to the results.

## Conclusion

The new ESPEN diagnostic criteria of malnutrition is a reliable tool to predict long-term mortality in geriatric inpatients. However, these results on mortality do not preclude the value of both definitions (ESPEN and MNA) on other important clinical outcomes. Therefore, more prospective studies are warranted to evaluate the association between the new ESPEN definition of malnutrition and other clinical outcomes (e.g. length of stay in the hospital, the risk of falls, functional impairment, and quality of life, etc.) in various populations.
